# Structure of a V_H_H isolated from a naïve phage display library

**DOI:** 10.1186/s13104-019-4197-0

**Published:** 2019-03-19

**Authors:** Brandy White, Ian Huh, Cory L. Brooks

**Affiliations:** Department of Chemistry, California State University Fresno, 2555 E San Ramon Ave, Fresno, CA 93740 USA

**Keywords:** Nanobody, V_H_H, Single domain antibody

## Abstract

**Objective:**

To determine the X-ray structure and biophysical properties of a Camelid V_H_H isolated from a naïve phage display library.

**Results:**

Single domain antibodies (V_H_H) derived from the unique immune system of the *Camelidae* family have gained traction as useful tools for biotechnology as well as a source of potentially novel therapeutics. Here we report the structure and biophysical characterization of a V_H_H originally isolated from a naïve camelid phage display library. V_H_H R419 has a melting temperate of 66 °C and was found to be a monomer in solution. The protein crystallized in space group *P*6_5_22 and the structure was solved by molecular replacement to a resolution of 1.5 Å. The structure revealed a flat paratope with CDR loops that could be classified into existing canonical loop structures. A combination of high expression yield, stability and rapid crystallization might make R419 into a candidate scaffold for CDR grafting and homology modeling.

## Introduction

The immune system of the *Camelidae* family (camels, llamas and alpacas) are unusual as in addition to possessing prototypic antibodies; their sera contain a species of antibody that has lost the light chain [1]. The variable domain of these *Camelidae* antibodies can be cloned, resulting in the smallest known functional antigen-binding unit—the V_H_H (also called nanobodies, single domain antibodies, or sdAb). V_H_H antibody fragments are small (13–15 kDa), heat stable, readily produced in *E. coli* and display distinct antigen-binding properties compared to traditional antibody fragments [2]. The isolation of antigen specific V_H_H is carried out using phage display from *Camelidae* immunization, naïve immune repertoires, or synthetic/semi-synthetic libraries [3].

The genes coding for the *Camelidae* heavy chain antibodies diverged from other ungulates 25 million years ago and have evolved sequence and structural differences which make them distinct from conventional hetero-dimeric antibodies [4]. Of particular note are the significant differences in the antigen binding complementarity determining region (CDR) loops CDR loops. V_H_H have been found to have unusually long CDR1 and CDR3 loops [5] and CDR1 and CDR2 canonical frequently depart from the canonical structures found in traditional antibodies [6].

The differences in V_H_H biophysical properties compared to traditional antibody formats have generated considerable interest in employing V_H_H for therapeutics, diagnostics and even for the detection of environmental pollutants [7–9]. Given the potential biotechnological and biomedical importance of V_H_H, the ability to construct highly accurate homology models is extremely useful. However, the differences in CDR structure can make structural homology modeling of V_H_H challenging [10]. The more V_H_H structures available will increase the success of modeling algorithms, and our understanding of the structural diversity present in V_H_H.

The V_H_H R419 was originally isolated from a pre-immune phage display library to bind the *Listeria* surface antigen Internalin B [11]. V_H_H R419 was later found to be non-functional as it was unable to bind InlB or inhibit *Listeria* invasion in vitro [12]. Here we report the structure and biophysical characterization of V_H_H R419 for its potential future value in homology modeling of other more therapeutically relevant V_H_H. The high yield, stability and ease of crystallization of R419 may make it into a valuable scaffold for CDR grafting.

## Main text

### Methods

#### Protein purification

**Fig. 1:**
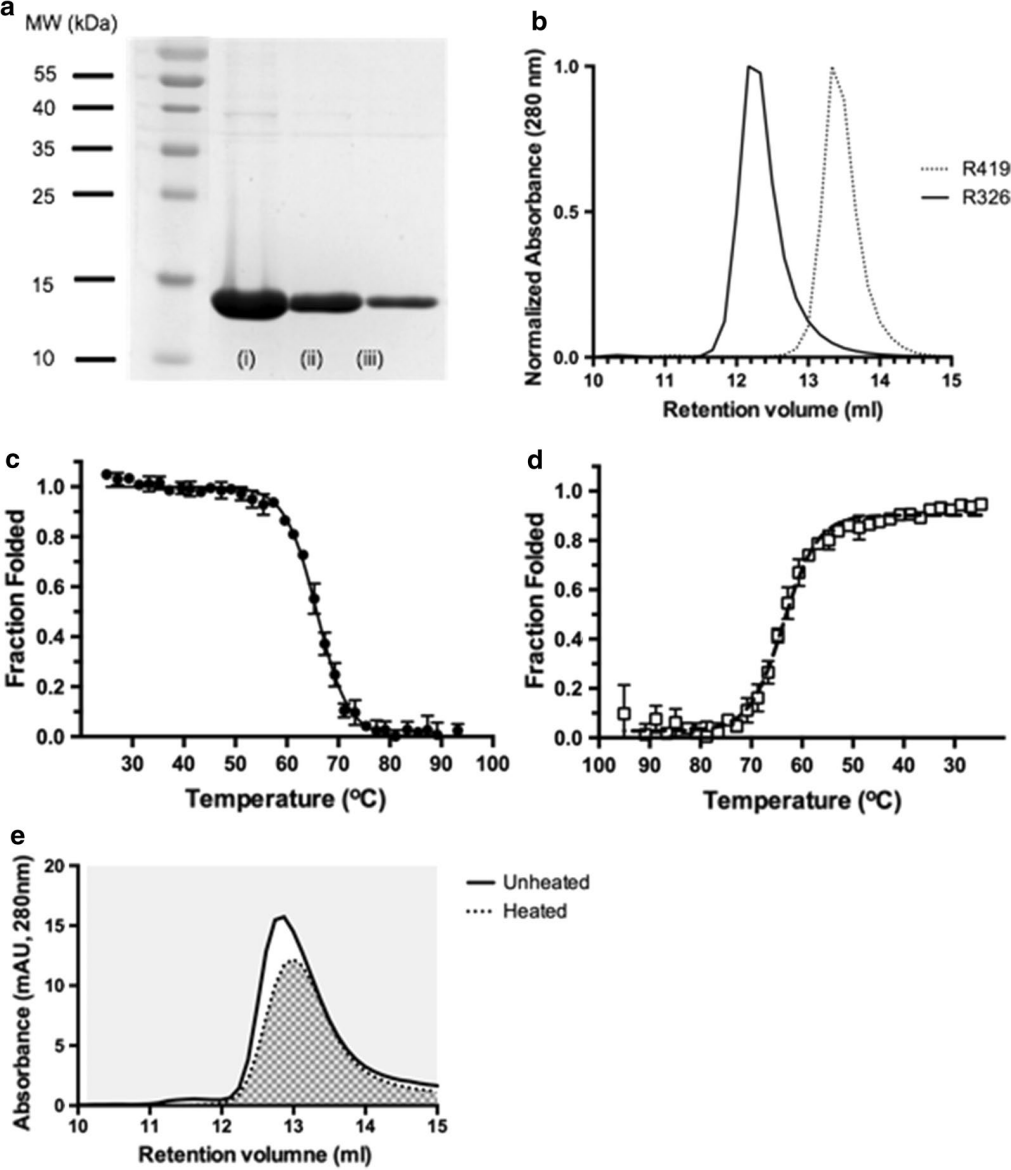
Purification and characterization of V_H_H R419. **a** SDS-PAGE of V_H_H R419 purification using immobilized metal affinity chromatography. The three lanes (i–iii) correspond to imidazole concentrations of the elutions (0.25 M, 0.5 M and 1 M). **b** Analytical size exclusion chromatography of V_H_H R419 (dashed line) and V_H_H R326 (solid line). Despite having the same molecular weight (14.7 kDa), the proteins display different elution volumes. **c** Thermal stability (Tm = 66 °C) of R419 measured by CD spectroscopy. **d** Reversible refolding of R419 measured by CD spectroscopy. Samples were cooled back to 25 ºC immediately following thermal denaturation. **e** Aggregation propensity of R419 during refolding was measured by size exclusion chromatography (SEC). Unheated R419 was injected onto a analytical SEC column. R419 was heated, cooled to allow refolding, centrifuged to remove aggregates and injected onto a SEC column. The difference of the areas under the curves represents the amount of sample unfolded and lost due to aggregation

The gene for V_H_H R419 [11] was codon optimized for *E. coli* expression and produced as a double stranded GenPart DNA fragment (Genscript Inc, NJ). The DNA fragment was cloned into the periplasmic expression vector pET-22b (EMD Millipore, USA). The plasmid was transformed into *E. coli* BL21 (DE3). An overnight culture was used to inoculate 2 × YT media. The culture was grown to mid-log phase (30 °C, 225 rev min^−1^, OD_600_ = 0.7) and induced with IPTG (0.4 mM). Fermentation was carried out overnight (16 h, 30 °C, 225 rev min^−1^). V_H_H R419 was extracted from the periplasm using an osmic shock procedure. Cells were harvested by centrifugation (5000*g*, 4 °C, 10 min) and suspended in ice cold TES buffer (0.2 M Tris pH 8.0, 0.5 M sucrose, 0.5 mM EDTA). The cells were mixed on ice for 30 min and an equal volume of ice-cold water was added and mixed on ice for an additional 30 min. The periplasmic fraction containing the V_H_H was harvested from the cell pellet (12,000*g*, 4 °C, 30 min) and dialyzed for an hour against 50 mM Tris pH 8.0, 0.15 M NaCl. The protein was purified using IMAC (immobilized metal-ion affinity chromatography) by batch binding the dialyzed periplasmic fraction to 1 ml of His-Pur Ni-NTA rein (Thermo Scientific, USA) for 1 h at 4 °C. Unbound protein was removed by washing with 30 column volumes of wash buffer (50 mM Tris pH 8.0, 0.3 M NaCl, 10 mM imidazole). The protein was eluted using a step gradient consisting of 0.25, 0.5 and 1 M imidazole. Protein purity was assessed by SDS-PAGE (Fig. 1a).

#### Analytical size exclusion chromatography

A size exclusion column (Enrich SEC70, BioRad) was equilibrated 2 CV of buffer (50 mM Tris–HCl (pH = 8), 0.15 M NaCl). R419 (0.2 ml, 1 mg ml^−1^) was injected onto the pre-equilibrated size exclusion column (flow rate of 0.5 ml min^−1^) protein elution was monitored at 280 nm (Fig. 1b). For molecular weight estimation, gel filtration standards ranging from 1.3 to 670 kDa (BioRad Inc.) were injected onto the size exclusion column. A standard curve was constructed and the molecular weight of R419 estimated from the curve.

#### Circular dichroism spectroscopy

R419 was diluted to 7.5 μM in PBS buffer and filtered through 0.45 μm microfilter. Using a JASCO J-815 CD spectrometer, a variable temperature measurement program was run at a wavelength of 200 nm from 25 to 95 °C. Reversible folding was monitored in the same sample by reversing the denaturation curve from 95 to 25 °C. The experiment was repeated three times, and the data exported to GraphPad Prism for final analysis.

#### Aggregation assay

The propensity of R419 to aggregate was measured by size exclusion chromatography. Samples of R419 (45 μg) were heated (80 °C, 10 min) and then cooled (4 °C, 10 min). Samples were then centrifuged (17,000×*g*, 10 min) and analyzed on an analytical size exclusion column (Enrich SEC70, BioRad). The amount of protein aggregation was expressed as the percent recovery of the area under the curve after heating relative to the unheated sample.

#### Crystallization

V_H_H R419 was dialyzed against 10 mM Tris pH 8.0, 30 mM NaCl and concentrated to 10 mg ml^−1^. Crystal screening was carried out in 96-well Intelli-plates (Hampton Research, USA) using the PEGs and PEG II crystal screens (Qiagen, USA). Diamond shaped crystals were observed in multiple conditions in both screens. Crystal conditions used for X-ray diffraction were 0.2 M ammonium sulfate, 0.1 M MES pH 6.5, 30% PEG 5000 monomethyl ether.

#### X-ray data collection and processing

Crystals of R419 were soaked in cryoprotectant (mother liquor containing 25% glycerol) and flash-frozen in liquid nitrogen. X-ray data was collected on beamline 08ID-1 at the Canadian Light Source equipped with a Rayonix MX300 CCD X-ray detector [13]. The data were processed with xia2 [14] (Table 1).

**Table 1:** X-ray data collection, processing and refinement

Parameter	V_H_H R419 (PDB Code 6DYX)
Diffraction source	CLSi beamline 08ID-1
Wavelength (Å)	0.98
Temperature (K)	100
Space group	*P*6_5_22
*a*, *b*, *c* (Å)	58.28, 58.28, 155.36
α, β, γ (°)	90, 90, 120
Resolution range (Å)	36.145–1.500 (1.540–1.500)
Total no. of reflections	259,791 (16,945)
No. of unique reflections	25,681 (2333)
Completeness (%)	99.200 (91.500)
Redundancy	10.100 (7.100)
〈*I*/σ(*I*)〉	10.700 (5.05)
*R*_r.i.m._	0.218 (0.359)
*R*_p.i.m._	0.067
Overall *B* factor from Wilson plot (Å^2^)	19.660
Resolution range (Å)	36.145–1.500 (1.5601–1.5000)
Completeness (%)	99.0
No. of reflections	25,678 (2455)
Final *R*_cryst_	0.176 (0.1854)
Final *R*_free_	0.190 (0.1977)
No. of non-H atoms	
Protein	853
Ion	14
Water	141
Total	1008
R.m.s. deviations	
Bonds (Å)	0.007
Angles (°)	0.875
Average *B* factors (Å^2^)	
Protein	23.3
Ion	49.2
Ligand	0.0
Water	40.1
Ramachandran plot	
Most favored (%)	100.00
Allowed (%)	0

Numbers in brackets represent values from the highest resolution shell

#### Structure solution and refinement

The structure of R419 was solved by molecular replacement using Phaser [15] as implemented in Phenix [16]. The V_H_H R303 (PDB code: 6DBA) was used as a search model [12]. Model building was carried out using Coot [17]. Final model statistics are provided in Table 1.

### Results and discussion

The V_H_H R419 expressed to a high yield in *E. coli* (~ 5 mg l^−1^ of culture) and was purified to homogeneity using a single step nickel affinity chromatography step from the *E. coli* periplasm (Fig. 1a). Biophysical characterization of purified V_H_H R419 was carried out using a combination of analytical size exclusion chromatography (SEC) and circular dichroism spectroscopy. The protein eluted from the SEC column as a single, monodisperse peak with a retention volume of 14.4 ml (Fig. 1b). To determine the quaternary structure of R419 from the SEC data, the molecular weight was calculated from a standard curve. Surprisingly, the molecular weight determined by SEC was determined to be only 5 kDa, which is ~ 3 times smaller that the molecular weight calculated form the sequence (14.7 kDa). A V_H_H of similar size to R419 (R326, 14.7 kDa), did not display this behavior (Fig. 1b, retention volume, 12 ml, calculated molecular weight 12 kDa). The rapid migration of R419 through the SEC column may be indicative of a compact structure and a monomeric configuration.

The stability, reversible folding and aggregations propensity of R419 was determined by a combination of circular dichroism (CD) spectroscopy, and by SEC. The thermal stability of R419 was measured by monitoring the thermal induced denaturation of the antibody (Fig. 1c). The melting temperature (T_m_) was calculated to be 66 °C from the denaturation curve. This places the stability of R419 well within the typical T_m_ range of 50–80 °C found in V_H_Hs [18]. One of the hallmark biophysical properties observed in some camelid V_H_H is reversible refolding following heating [19]. The aggregation and refolding properties of R419 were examined using several approaches. First, CD spectroscopy was used to follow the refolding of R419. Immediately following heat denaturing (Fig. 1c), the temperature gradient was reversed and the sample was cooled to allow for refolding (Fig. 1d). Nearly the entire sample was refolded, with 85–90% of the original CD signal being restored upon cooling (Fig. 1d). For another quantitative assessment of in solution refolding, R419 was heated and the recovery of the sample after cooling was calculated after injection onto a SEC column which readily separates aggregates [20]. A percent recovery of 82% (± 2%) was calculated from four experiments (Fig. 1e). These results suggest that R419 displays superior biophysical properties in terms of reversible folding and aggregation resistance. Aggregation resistance and reversible folding in camelid V_H_H domains was believed to be a hallmark of the domain that distinguished them from the homologous V_H_3 domains found in human antibodies [19]. However, a recent survey of 70 camel and llama V_H_H found that aggregation resistance and reversible folding were very rare, with only 1–5% of antibodies reversibly folding [21]. Interestingly, R419 contains none of the factors correlated with the propensity of V_H_H to reversibly fold, such as an usually long CDR3 loop (see below), or the presence of a non-canonical disulfide bond [21].

**Fig. 2:**
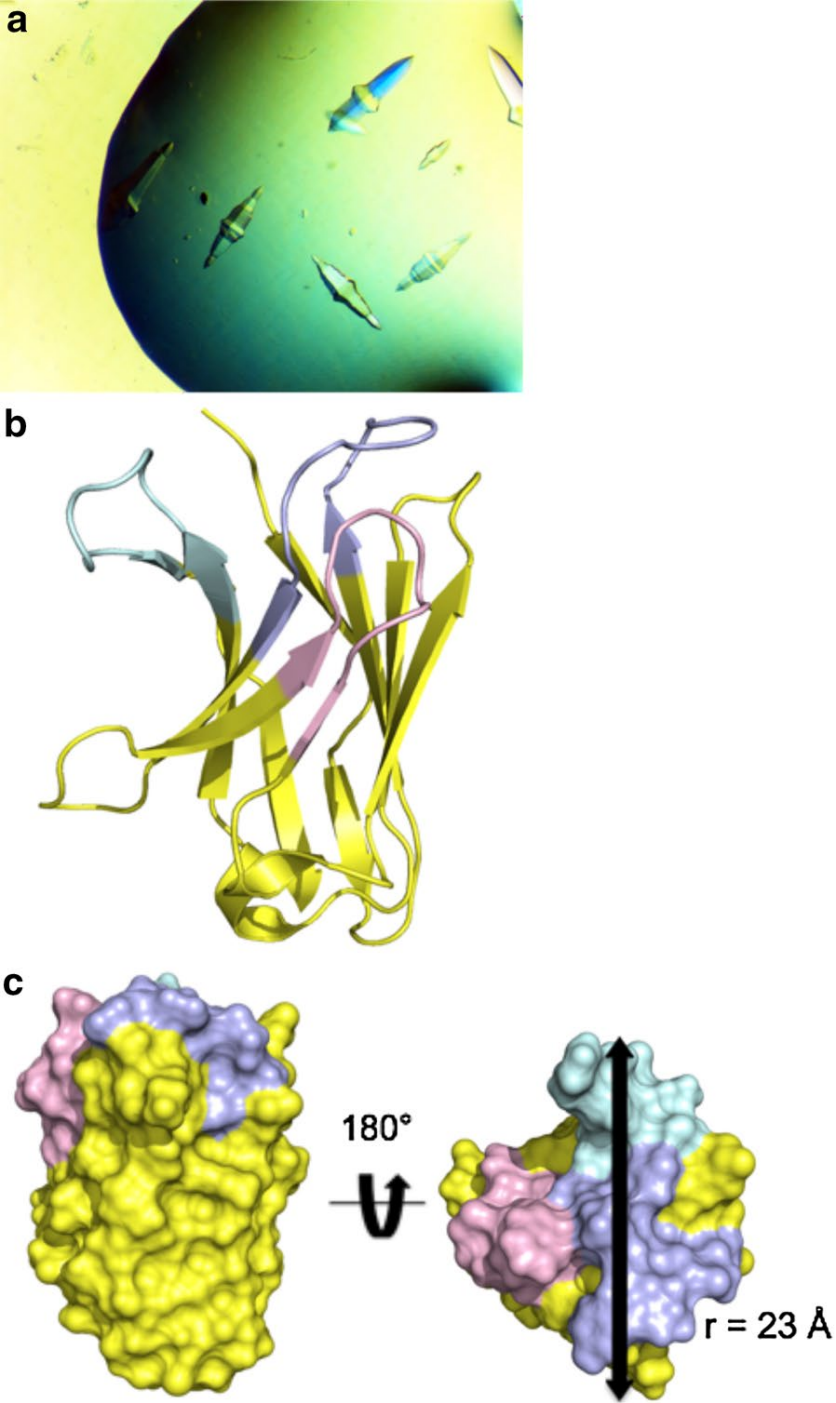
Crystallization and structure D of V_H_H R419. **a** Crystals of R419 grown in PEG MME and MES buffer. **b** Ribbon structure of R419. CDR loops are colored, CDR 1—violet, CDR 2—light pink, CDR 3—cyan. **c** Paratope structure of V_H_H R419. The paratope of R419 is flat and has a radius of 23 Å

R419 was an easily crystallized V_H_H. Well-formed, diamond shaped crystals appearing directly in robotic screens in several conditions within a few days (Fig. 2a). Similar appearing crystals grew in a total of 10 conditions. These conditions contained PEG 4000 or PEG 5000 MME, with Tris, HEPES and MES buffer (pH 6.5–8.5). Crystals from the robot screen diffracted to a resolution of 1.5 Å, and the structure was readily solved by molecular replacement (Table 1). The structure contained a single molecule in the asymmetric unit, with no major disordered regions (Fig. 2b).

In contrast to the flat or convex paratope shape associated with traditional antibodies, the paratope region of V_H_H are typically classified as being either flat or convex [6]. The paratope of R419 is flat in shape, with a radius of 23 Å, which falls within the normal distribution of V_H_H paratope radii of 15–25 Å [6] (Fig. 2c).

The CDR loops of R419 were classified using the standard canonical clusters as described by North [22]. CDR 1 is 13 amino acids long and falls within the 4th cluster (Fig. 2b). While 13 amino acids is the most common loop length for CDR H1 in human, mice, as well as camelid species, cluster 4 is only found in 2.6% of alpaca CDR1 loops, 0.8% of Llama CDR1 loops [6, 22]. The CDR2 loop of R419 is 10 amino acids long, and falls within cluster 2, which is the most common canonical structure observed in camelid V_H_H (Fig. 2c). CDR3 is 11 amino acids long, which is within the range falls within the median loop range of 7–16 residues found within 86% of antibody structures [22].

In many ways V_H_H R419, is an “average” V_H_H, with biophysical and structural properties typical of many camelid V_H_H structures. The structure presented here may be useful for homology modeling of similar V_H_H or given the protein’s high expression yield, mid-range thermal stability, reversible folding lacking in significant aggregation and ease of crystallization, R419 may be a valuable tool for CDR scaffolding or as the basis of a novel semi-synthetic phage display library for VHH discovery.

## Limitations

- VHH R419 has no direct therapeutic or diagnostic value as it does not appear to bind the InlB antigen.
- We have yet to demonstrate that VHH R419 is a valuable tool for CDR grafting.

## Abbreviations

V_H_H: variable region from camelid single domain heavy chain antibody
CDR: complementarity determining region
SEC: size exclusion chromatography
CD: circular dichroism
